# An evaluation of antenatal corticosteroid use and timing in a large US birth cohort 2017–2021

**DOI:** 10.1002/pmf2.70287

**Published:** 2026-04-07

**Authors:** Vivienne Souter, C. Andrew Combs, Ian Painter, Kristin Sitcov, Lisa Foglia, Sarah M. Edwards, Asma Khalil, Andreas Hoehn

**Affiliations:** ^1^ Obstetrical Care Outcomes Assessment Program (OB COAP) Foundation for Health Care Quality Seattle Washington USA; ^2^ Public Health, School of Health and Wellbeing University of Glasgow Glasgow Scotland UK; ^3^ The Pediatrix Center for Research Education, Quality and Safety Sunrise Florida USA; ^4^ Maternal Fetal Medicine Mountain Area Health Education Center Asheville North Carolina USA; ^5^ Gynecologic Surgery & Obstetrics Uniformed Services University Bethesda Maryland USA; ^6^ CommonSpirit Southern Colorado Maternal Fetal Medicine Colorado Springs Colorado USA; ^7^ Vascular Biology Research Centre Molecular and Clinical Sciences Research Institute City St George's University of London London UK

**Keywords:** antenatal corticosteroids, late preterm steroids, preterm birth

## Abstract

**Introduction:**

There is a lack of published data on the contemporary use and timing of antenatal corticosteroids in the United States. Our primary objective was to address this gap using clinical data from a large US birth cohort. Our secondary objective was to identify factors associated with optimally timed steroids in births <34 weeks’ gestation.

**Methods:**

This retrospective study included de‐identified clinical data from a quality improvement database for births at 24^+0^–42^+6^ weeks’ gestation between January 1, 2017 and December 31, 2021 at 12 hospitals in the northwest United States. We evaluated the proportion of live births <34 weeks’ gestation that received at least a first dose of antenatal steroids within the 7 days preceding birth (“optimally timed” steroids), and the proportion of patients given antenatal steroids who delivered at term. Annual rates of these measures were also evaluated across the study period. Associations between optimal steroid timing in preterm births <34 weeks’ gestation and sociodemographic and medical characteristics were explored with a multilevel logistic regression model using hospitals as random effects.

**Results:**

Of 105,981 patients included in the analysis, 6.1% (6443) received steroids before 37 weeks’ gestation. A total of 83.1% of live births <34 weeks’ gestation received antenatal steroids, but only 55.4% received optimally timed steroids. The rate of optimally timed steroids in births <34 weeks’ gestation was stable between 2017 and 2021. Over the same period, the proportion of births at 34^+0^–36^+6^ weeks’ gestation that received steroids increased from 36.6% to 46.6%, and the proportion that received steroids and then delivered at term increased from 15.5% to 22.1% (*p* < 0.001). After adjusting for confounders, preeclampsia was associated with increased odds for optimally timed steroids in births <34 weeks’ gestation (OR 1.46; 95% CI 1.20, 1.79) while ruptured membranes (odds ratio [OR], 0.76; 95% CI, 0.63, 0.91) and placenta previa (OR, 0.28; 95% CI, 0.16, 0.51) were associated with decreased odds.

**Conclusion:**

In our study, most patients who delivered at <34 weeks’ gestation received at least one dose of antenatal corticosteroids, but this was outside of the optimal 7‐day window in approximately half of cases. Quality improvement efforts should focus on timing, as well as the use of antenatal corticosteroids.

## 1 INTRODUCTION

Antenatal corticosteroids (ACS) are one of the most efficacious interventions in obstetrics, reducing neonatal morbidity and mortality in births occurring before 34 weeks’ gestation (early preterm births) and neonatal respiratory morbidity in births at 34^+0^–36^+6^ weeks’ gestation (late preterm births) [1]. In addition to clear benefit when given between 1 and 7 days before birth, antenatal steroids might also have benefits when given as long as 14 days before birth [1, 2, 3]. However, timing of steroids is challenging due to the limited ability to predict the timing of preterm birth. Clinicians’ decisions, therefore, must balance potentially missing the opportunity to give ACS before a preterm birth with the possibility of giving them too early or unnecessarily if the pregnancy continues to term.

Studies using US birth certificate data have reported an increase in steroid use in both preterm and term neonates since the publication of the Antenatal Late Preterm Steroids (ALPS) Trial in 2016 [4, 5, 6, 7]. However, birth certificates do not record the gestational age when steroids are given and might also underascertain their use [5]. In 2022, the Society for Maternal‐Fetal Medicine (SMFM) published a statement on “Quality metrics for optimal timing of antenatal corticosteroid administration” which aimed to encourage more judicious use of steroids and outlined a quality metric for optimal timing defined as the proportion of patients with a livebirth between 24^+0^ and 33^+6^ weeks’ gestation who received at least one dose of steroids between 6 h and 7 days before birth [8]. However, there remains a lack of published clinical data on the contemporary use and timing of steroids in early preterm and late preterm births in the United States. Our primary objective was to address this gap using clinical data collected for quality improvement in a large and representative birth cohort. Our secondary objective was to identify factors associated with the use of steroids in the 7 days preceding births at less than 34 weeks’ gestation.

## 2 MATERIALS AND METHODS

This retrospective study used clinical data from a perinatal quality improvement organization in the northwest United States: Obstetrical Care Outcomes Assessment Program (OB COAP), which is part of the Foundation for Health Care Quality in Seattle, WA, USA. OB COAP acquires data from the medical records of all births ≥20 weeks’ gestation at participating sites through chart abstraction by trained data managers and by direct uploading of select fields from electronic medical records, where possible. Definitions of each data element are provided at the point of data entry and, where applicable, are based on standardized American College of Obstetricians and Gynecologists (ACOG) definitions including those provided in “reVITALize” [9]. Quality checks are performed at data entry, at the site level, and at the aggregate level. Data are deidentified before analysis for research.

The study included 12 hospitals that contributed data to OB COAP throughout the study period (January 1, 2017 to December 31, 2021). Eleven hospitals were in Washington state and one was in Montana. Three of the hospitals provided level I neonatal care, four provided level II neonatal care, and five provided level III or IV neonatal care; as defined by the American Academy of Pediatrics, level I being the most basic level of care and level IV being the highest level [10].

Inclusion criteria were singleton or multifetal births at 24^+0^–42^+6^ weeks of gestation. Pregnancies with missing data on ACS administration or the timing of ACS were excluded, as were 45 pregnancies that were recorded as receiving ACS after 37 weeks’ gestation. The unit of analysis was pregnancy (not neonate), so a twin pregnancy was counted only once.

The primary outcomes were administration of steroids and the timing of the first dose relative to the timing of birth and the percentage of pregnancies given steroids that ultimately delivered at term. The dataset recorded only the day of steroid administration, not the hour, and therefore, timing before birth was to the nearest day. If steroids were given at another hospital or as an outpatient, abstractors were asked to record this from prenatal records or transfer records. We distinguished preterm birth as early preterm (defined as birth at < 34 weeks’ gestation) and late preterm (defined as birth at 34^+0^–36^+6^ weeks’ gestation).

Data on sociodemographic and health risk profiles included maternal age, prepregnancy diabetes, prepregnancy hypertension, maternal weight, smoking during pregnancy, illicit substance use during pregnancy, antenatally detected fetal anomalies, and preeclampsia or gestational hypertension. Race, as reported in the medical chart, was also included as racial disparities in steroid use have been previously reported [11, 12]. Body mass index was the final BMI that was recorded closest to the date of delivery; values < 15 kg/m^2^ or > 80 kg/m^2^ were considered implausible and categorized as missing. Parity was categorized as nulliparous, parous with no prior preterm birth, and parous with a history of preterm birth.

Health insurance status was categorized as commercial versus government‐funded. We included an area‐level marker of socioeconomic status, the Economic Innovation Group's Distressed Community Index (DCI) (https://eig.org/distressed‐communities/) [13]. The DCI summarizes the prosperity of a patient's zip code of residence using various parameters, such as median household income, the prevalence of unemployment, and poverty, and is expressed as quintiles: prosperous, comfortable, mid‐tier, at risk, and distressed. The type of health care professional (e.g., family practitioner, obstetrician, midwife, maternal‐fetal medicine specialist) responsible for the patient's medical care during the prenatal period was also evaluated.

Overall, levels of completeness in the data were high with less than 9% missing for any variable in the ACS or non‐ACS groups.

We evaluated the proportion of all preterm live births that received at least one dose of steroids and stratified this metric by early preterm birth and late preterm birth. We tabulated the timing of the first dose as: within the 7 days before birth; < 1 day before birth; 1–7 days before birth; 8–14 days; and ≥ 15 days before birth. Timing of steroids was stratified for early preterm birth, late preterm births, and all preterm births. We also plotted the median interval (in days) between the first dose of steroids and delivery, by gestational week at the first dose.

We also aimed to estimate the use and timing of second courses of ACS, acknowledging limitations in the data for this. Data on second courses of ACS and timing of antenatal steroids have been collected since 2017. Initially, some second *doses* of the first course of ACS were erroneously entered into the field for the first dose of the second *course* of steroids. Given this, in order to estimate the use of a second course of ACS, a data cleaning step had to be performed: only pregnancies where the second course of ACS was given at least 7 days after the date of the first dose of the first course were considered as having a second course.

To align with the SMFM quality measure for optimal steroid use, preterm metrics were restricted to pregnancies with live births while the percentage of pregnancies given steroids that delivered at term (the SMFM balancing metric) included both live births and stillbirths [8]. Optimal timing was defined as the first dose of steroids being given within the 7 days preceding preterm birth.

Univariate analysis was performed to evaluate any changes in annual rates in the metrics over time. Odds ratios (ORs) and 95% confidence intervals (95% CIs) were evaluated using 2017 as the reference year.

We used logistic multilevel models to explore associations between the covariates and optimal timing of the first dose of ACS in early preterm births [14]. Unconditional fixed effects were run for optimally timed steroid use in early preterm births, without any other variables in the models. We assumed a multilevel structure as clinician practices are clustered within hospitals and patient‐level characteristics might be more similar within hospitals than between hospitals [14, 15]. Multivariate multilevel logistic modeling was then run for optimally timed steroid use in early preterm births, including all relevant variables, and using hospitals as random intercepts. Variables were included in the multivariable model if they were significantly associated with optimally timed steroid use on univariate analysis, had previously been reported as factors in steroid use, or could plausibly be associated with steroid use. For any variables where data were missing for more than 100 births, these were included as a separate “missing” category in the models.

Hospital level of care was not included in the models because the hospital of birth was included as a cluster feature in the models as random intercepts [14]. Including hospitals in the model as random intercepts controls for potential differences between hospitals while also accounting for within‐hospital correlation (the cluster effect). This also acts to adjust for any level of care effects. All analyses were performed in Stata 14.2 (Stata Corp).

## 3 RESULTS

**FIGURE 1:**
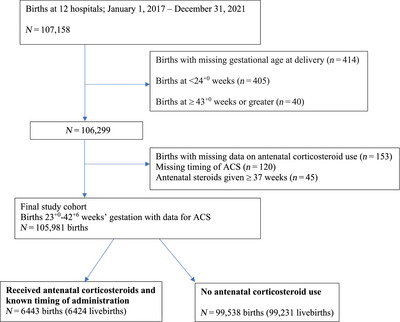
Study cohort. ACS, antenatal corticosteroids.

A total of 107,158 pregnancies at 12 hospitals during the study period were evaluated. After exclusion of pregnancies with missing gestational age at birth (*n* = 414), births at < 24 weeks (*n* = 405), births at ≥43 weeks (*n* = 40), births with missing data on steroid usage (*n* = 153) or timing of the first dose (*n* = 120), and births that were recorded to have had steroids at 37 weeks’ gestation or later (*n* = 45), 105,981 pregnancies were included in the final analysis (105,655 with live births and 326 with stillbirths) (Figure 1).

Preterm live birth (<37 weeks’ gestation) occurred in 9783 (9.2%) of the study cohort: 2641 (2.5%) were early preterm (<34 weeks’ gestation) and 7142 (6.7%) were late preterm (34^+0^–36^+6^ weeks) (Table 1).

**TABLE 1:** Characteristics of the study cohort.

	No antenatal steroids *N* = 99,538 *n* (%)	Antenatal steroids *N* = 6443 *n* (%)	Pearson chi2 test *p* value
Maternal age, years			*p* < 0.001
<20	2893 (2.9)	220 (3.4)	
20–24	14,034 (14.1)	877 (13.6)	
25–29	27,568 (27.7)	1675 (26.0)	
30–34	32,963 (33.1)	1920 (29.8)	
35–39	18,220 (18.3)	1358 (21.1)	
40–54	3849 (3.9)	390 (6.0)	
Missing	11 (0.01)	3 (0.05)	
Pregnancy history			*p* < 0.001
Nulliparous	39,725 (39.9)	2795 (43.4)	
Parous, *no* previous preterm birth	57,316 (57.6)	3056 (47.4)	
Parous, previous preterm birth	2497 (2.5)	592 (9.2)	
Health history			
Prepregnancy diabetes	1920 (1.9)	326 (5.1)	*p* < 0.001
Prepregnancy hypertension, *n* (%)	3572 (3.6)	868 (13.5)	*p* < 0.001
Final BMI (kg/m^2^)			*p* < 0.001
<25	8387 (8.4)	748 (11.6)	
25–29.9	31,406 (31.6)	1856 (28.8)	
≥30	56,235 (56.5)	3605 (56.0)	
Missing	3510 (3.5)	234 (3.6)	
Pregnancy complications			
Multiple pregnancy	1103 (1.1)	811 (12.5)	*p* < 0.001
Preeclampsia/gestational hypertension (irrespective of gestational age at diagnosis or onset), *n* (%)	11,076 (11.1)	1989 (30.9)	*p* < 0.001
Missing	91 (0.1)	6 (0.1)	
Placenta previa	258 (0.3)	219 (3.4)	*p* < 0.001
Fetal abnormality	1770 (1.8)	413 (6.4)	*p* < 0.001
Smoking during pregnancy	5279 (5.3)	512 (8.0)	*p* < 0.001
Missing	31 (0.03)	1 (0.02)	
Illicit substance use during pregnancy	2175 (2.2)	285 (4.4)	*p* < 0.001
Missing	28 (0.03)	1 (0.02)	
Gestation at birth (weeks)			
<34 (*n* = 2832)	620 (0.6)	2212 (34.3)	
34^+0^–36^+6^ (*n* = 7196)	4169 (4.2)	3027 (47.0)	
≥37 weeks (*n* = 95,953)	94,749 (95.2)	1204 (18.7)	
Stillbirth	307 (0.3)	19 (0.29)	0.849
Sociodemographic characteristics			
Race			*p* < 0.001
Hispanic or Latina	16,105 (16.2)	998 (15.5)	
Non‐Hispanic Asian	14,260 (14.3)	792 (12.3)	
Non‐Hispanic Black	5890 (5.9)	511 (7.9)	
Non‐Hispanic Hawaiian or Pacific Islander	1862 (1.9)	150 (2.3)	
Non‐Hispanic Native American or Native Alaskan	1436 (1.4)	148 (2.3)	
Non‐Hispanic White	51,016 (51.3)	3144 (48.5)	
Multiple racial groups or group not included above.	2824 (2.8)	159 (2.5)	
Missing	6145 (6.2)	561 (8.7)	
Distressed communities index (quintiles)			*p* < 0.001
Prosperous	35,904 (36.1)	2120 (32.9)	
Comfortable	27,260 (27.4)	1713 (26.6)	
Mid‐tier	13,761 (13.8)	1104 (17.1)	
At risk	16,272 (16.4)	1032 (16.0)	
Distressed	5014 (5.0)	358 (5.6)	
Missing, % (*n*)	1327 (1.3)	116 (1.8)	
Pregnancy care			
Intrapartum transfer into hospital	1169 (1.2)	791 (12.3)	*p* < 0.001
Prenatal care professional			*p* < 0.001
Family practitioner	9294 (9.3)	335 (5.2)	
Maternal fetal medicine specialist	2244 (2.3)	858 (13.2)	
Midwife	10,703 (10.8)	314 (4.9)	
Obstetrician	71,870 (72.2)	3947 (61.3)	
Other	3979 (4.0)	674 (10.5)	
Missing	1448 (1.5)	315 (4.9)	
Commercial insurance payor	58,618 (58.9)	3537 (54.9)	*p* < 0.001
Missing	1099 (1.1)	32 (0.5)	
Hospital level of neonatal care, *n* (%)			*p* < 0.001
I	7381 (7.4)	70 (1.1)	
II	24,409 (24.5)	785 (12.2)	
III–IV	67,748 (68.1)	5588 (86.7)	

Abbreviation: BMI, body mass index.

Antenatal steroids were given at some point during 6.1% (6443/105,981) of all pregnancies in the study and 1.3% (1204/95,953) of all term live births (Table 1 and Table S1). Pregnancies associated with prepregnancy maternal health issues, previous preterm birth, complications in the current pregnancy, and multiple pregnancies were overrepresented among the group who received steroids (Table 1). Of the 6443 pregnancies given steroids, 2212 (34.3%) delivered <34 weeks, 3027 (47.0%) delivered at 34^+0^–36^+6^ weeks, and 1204 (18.7 %) delivered at term (37 weeks’ gestation or later) (Table 1).

Of the 2641 early preterm births in the study, 2196 (83.2 %) received steroids at some point, 1466 (55.5%) received their first dose within the 7 days preceding birth, and 244 (9.2%) and 931 (35.3%) did not receive them or received them 15 days or more before giving birth (Table 2).

**TABLE 2:** Timing of first dose of antenatal corticosteroids (ACS).

Gestation at birth (weeks)^a^	No ACS given % (*n*)	ACS within 1 day of birth % (*n*)	ACS 1–7 days before birth % (*n*)	ACS 8–14 days before birth % (*n*)	ACS * ≥ *15 days before birth *n* (%)
**<34** *N* = 2641	16.9 (445)	13.3 (351)	42.2 (1115)	9.2 (244)	18.4 (486)
**34^+0^–36^+6^** *N* = 7142	57.7 (4118)	6.9 (493)	20.4 (1.454)	5.0 (366)	10.0 (711)
**<37** *N* = 9783	46.6 (4563)	8.6 (844)	26.3 (2569)	6.2 (610)	12.2 (1197)

^a^ Includes live births only.

Of the 7142 late preterm births, 3024 (42.3%) received steroids at some point, 1947 (27.3%) received their first dose within the 7 days preceding birth, and 4829 (67.6%) did not receive them or received them more than 14 days before giving birth (Table 2). For all preterm live births (<37 weeks’ gestation), 5220 (53.4%) received steroids and 3413 (34.9%) received their first dose within the 7 days preceding birth (Table 2).

**FIGURE 2:**
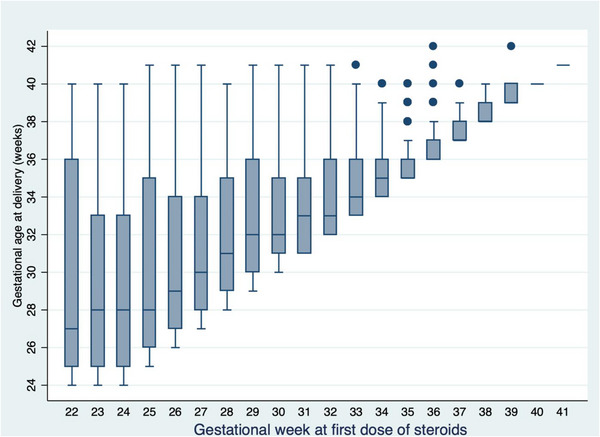
Median gestational age at delivery (weeks) by gestational week at first dose of antenatal corticosteroids. The horizontal line in the box plot indicates the median gestation at delivery in weeks, the upper limit of the box plot indicates the 75th percentile, and the lower limit of the box plot indicates the 25th percentile.

Of pregnancies given steroids at less than 34 weeks’ gestation, 55.5% delivered early preterm, 27.3% late preterm, and 17.2% at term (Appendix S1 and Table S2). The median interval between the first dose of steroids and delivery was 6 days overall, decreasing from 39 days for those given at 22 weeks’ gestation to 2 days at 36 weeks’ gestation (Figure 2).

Using the data cleaning approach described in Section 2, a second course of ACS was identified in 229 (8.7%) of the 2641 patients who had a livebirth <34 weeks’ gestation. Of these, 167 (72.9%) gave birth within 7 days of initiation of the second course of steroids. Considering all 2641 early preterm births, 1466 delivered within 7 days of the first dose of ACS and 167 within 7 days of the second course of ACS, consistent with 1633 (61.8%) of all births at <34 weeks’ gestation occurring within 7 days of ACS.

The annual rate of optimally timed steroids in early preterm births remained stable across the study period (Table 3). The percentage of pregnancies that were given steroids and delivered at term increased from 15.5% in 2017 to 22.1% in 2021 (OR, 1.55; 95% CI, 1.26–1.89; *p* < 0.001) (Table 3) and the percentage of term live births given steroids at some point during pregnancy increased from 0.96% in 2017 to 1.57% in 2021 (Appendix S1 and Table S1). The percentage of late preterm births that were given steroids increased from 36.6% in 2017 to 46.0% in 2021, and the percentage of late preterm births that received a first dose of steroids within 7 days of birth increased from 23.3% in 2017 to 29.6% in 2021 (Table 4). By 2021, 41.7% of all steroids were administered during the late preterm period (Appendix S1 and Table S3). Notably, the preterm birth rate for the entire study cohort was 9.5% (*n* = 10,033) and was stable across the study period.

**TABLE 3:** Annual proportion of early preterm births (< 34 weeks’ gestation) given a first dose of antenatal corticosteroids (ACS) within 7 days of birth (optimal timing) and the proportion of births given ACS that delivered at term (37 weeks’ gestation or later).

Year of birth	% (*n*/*N*) of live births at < 34 weeks’ gestation given ACS within 7 days	Odds ratio (95% CI)	% (*n*/*N*) of births given ACS that delivered at term	Odds ratio (95% CI)
2017	58.0 (335/578)	Reference	15.5 (187/1207)	Reference
2018	54.3 (303/558)	0.86 (0.68, 1.09)	18.8 (238/1267)	1.26 (1.02, 1.56)
2019	55.6 (291/523)	0.91 (0.72, 1.16)	16.6 (205/1234)	1.09 (0.88, 1.35)
2020	54.8 (261/476)	0.88 (0.69, 1.12)	19.8 (267/1346)	1.35 (1.10, 1.66)
2021	54.5 (276/506)	0.87 (0.68, 1.11)	22.1 (307/1389)	1.55 (1.26, 1.89)
All years	55.5 (1466/2641)	–	18.7 (1204/6443)	

*Note*: The Pearson Chi‐2 *p* value was 0.732 for the % of early preterm livebirths given steroids within 7 days and < 0.001 for the % of births given steroids that delivered at term over time.

Abbreviation: CI, confidence interval.

**TABLE 4:** Annual proportion of late preterm births (34^+0^–36^+6^) given any antenatal corticosteroids (ACS) and the proportion given a first dose within 7 days of delivery.

Year	% (*n*) of live births at 34^+0^–36^+6^ weeks’ gestation given ACS	Odds ratio (95% CI)	% (*n*) of live births at 34^+0^–36^+6^ weeks’ gestation given ACS within 7 days	Odds ratio (95% CI)
2017 *N* = 1441	36.6% (527)	Reference	23.3% (335)	Reference
2018 *N* = 1413	40.2% (568)	1.17 (1.00, 1.36)	25.8% (364)	1.15 (0.97, 1.36)
2019 *N* = 1425	41.2% (587)	1.21 (1.05, 1.41)	26.1% (372)	1.17 (0.98, 1.38)
2020 *N* = 1398	47.8% (668)	1.59 (1.37, 1.84)	31.9 (443)	1.53 (1.17, 1.64)
2021 *N* = 1465	46.0% (674)	1.48 (1.27, 1.71)	29.6 (433)	1.39 (1.17, 1.64)
All *N* = 7142	42.3% (3024)	–	27.3% (1947)	–

*Note*: The Pearson Chi‐2 *p* value was < 0.001 for both comparisons over time.

Abbreviation: CI, confidence interval.

To identify factors associated with optimally timed steroid administration among early preterm births, characteristics of early preterm pregnancies were stratified by optimal versus suboptimal steroid timing (Appendix S1 and Table S4). In univariable analyses, no statistically significant differences were detected in optimally timed steroids by race, health insurance type (commercial vs. other), or DCI status. Preeclampsia was associated with a higher rate of optimal timing, while ruptured membranes, placenta previa, and illicit substance use were associated with lower rates. Rates of optimally timed steroids also varied by the type of obstetric professional providing prenatal care with maternal‐fetal medicine (MFM) specialists having the lowest rate (48.2%).

After multivariable adjustment for the covariates (Table 5), factors associated with significantly increased odds of optimally timed steroid use in early preterm births were nulliparity (OR, 1.23; 95% CI, 1.01, 1.48), multiple or other race (OR, 1.87; 95% CI, 1.07, 3.28), preeclampsia (OR, 1.46; 95% CI, 1.20, 1.79), and transfers into the hospital (OR, 2.69; 95% CI, 2.14, 3.37). Factors associated with significantly decreased odds of optimal timing were ruptured membranes at the time of admission (OR, 0.76; 95% CI, 0.63, 0.91), placenta previa (OR, 0.28; 95% CI, 0.16, 0.51), and prenatal care by an MFM specialist (OR, 0.69; 95% CI, 0.54, 0.87), likely due to their complex high‐risk patient population.

**TABLE 5:** Multilevel mixed effects logistic regression to evaluate factors associated with receiving antenatal corticosteroids within 7 days in early preterm live births (<34 weeks).

	Odds ratio (OR) for optimally timed steroids in early preterm live births OR (95% CI)	Adjusted odds ratio (aOR) for optimally timed steroids in early preterm live births aOR (95% CI)
Maternal age, years		
<20	1.09 (0.73, 1.62)	0.91 (0.58, 1.42)
20–24	1.18 (0.91, 1.51)	1.05 (0.79, 1.41)
25–29	0.92 (0.76, 1.34)	0.87 (0.70, 1.09)
30–34	Reference	Reference
35–39	0.89 (0.72, 1.10)	0.89 (0.71, 1.12)
40–54	0.80 (0.57, 1.12)	0.83 (0.57, 1.21)
Final BMI (kg/m^2^)		
<25	0.82 (0.65, 1.03)	0.93 (0.72, 1.20)
25–29	0.97 (0.82, 1.16)	1.09 (0.90, 1.33)
≥30	Reference	Reference
Missing	0.76 (0.56, 1.04)	0.94 (0.66, 1.34)
Birth history		
Parous, *no* previous births	Reference	Reference
Nulliparous	1.35 (1.16, 1.58)	1.23 (1.01, 1.48)
Parous, history of preterm birth	0.88 (0.67, 1.16)	0.90 (0.68, 1.20)
Health history		
Prepregnancy hypertension	1.21 (0.97, 1.50)	0.98 (0.76, 1.26)
Prepregnancy diabetes	1.18 (0.87, 1.59)	1.31 (0.93, 1.84)
Pregnancy complications		
Multiple pregnancy	1.06 (0.86, 1.30)	1.04 (0.83, 1.30)
Preeclampsia	1.96 (1.66, 2.13)	1.46 (1.20, 1.79)
Placenta previa	0.33 (0.19, 0.58)	0.28 (0.16, 0.51)
Fetal abnormality	0.51 (0.38, 0.68)	0.85 (0.60, 1.19)
Ruptured membranes at admission to Labor and Delivery	0.82 (0.70, 0.96)	0.76 (0.63, 0.91)
Smoking during pregnancy	0.77 (0.60, 0.99)	1.01 (0.74, 1.38)
Illicit substance use during pregnancy	0.66 (0.49, 0.89)	0.76 (0.52, 1.10)
Sociodemographic characteristics		
Race		
Non‐Hispanic White	Reference	Reference
Non‐Hispanic Black	1.00 (0.77, 1.32)	1.26 (0.93, 1.70)
Hispanic or Latina	1.07 (0.86, 1.33)	1.00 (0.78, 1.27)
Non‐Hispanic Asian	1.12 (0.86, 1.46)	1.17 (0.88, 1.55)
Non‐Hispanic Hawaiian or Pacific Islander	0.71 (0.42, 1.20)	0.78 (0.45, 1.38)
Non‐Hispanic Native American or Native Alaskan	0.72 (0.45, 1.17)	0.85 (0.51, 1.42)
Other or multiple race	1.40 (0.87, 2.24)	1.87 (1.07, 3.28)
Missing	1.05 (0.79, 1.39)	0.98 (0.72, 1.34)
Distressed communities index quintiles		
Prosperous	Reference	Reference
Comfortable	1.02 (0.82, 1.26)	0.91 (0.72, 1.13)
Mid‐tier	1.18 (0.92, 1.50)	1.03 (0.79, 1.34)
At risk	0.89 (0.69, 1.13)	0.85 (0.65, 1.10)
Distressed	0.88 (0.63, 1.24)	0.79 (0.54, 1.14)
Missing	0.77 (0.45, 1.31)	0.74 (0.41, 1.33)
Pregnancy care		
Commercial insurance payer	0.88 (0.76, 1.03)	0.91 (0.75, 1.10)
Pregnancy care		
Prenatal care professional		
Obstetrician	Reference	Reference
Family practitioner	1.17 (0.79, 1.73)	1.26 (0.83, 1.93)
Maternal fetal medicine specialist	0.77 (0.61, 0.98)	0.69 (0.54, 0.87)
Midwife	1.10 (0.75, 1.61)	1.27 (0.83, 1.94)
Other	1.12 (0.86, 1.44)	1.00 (0.77, 1.31)
Missing	1.09 (0.82, 1.44)	0.86 (0.63, 1.19)
Intrapartum transfer	2.84 (2.30, 3.51)	2.69 (2.14, 3.37)

Abbreviation: BMI, body mass index.

^a^ Adjustments include all variables in the table.

## 4 DISCUSSION

In this study of over 100,000 pregnancies, we found that 83% of live births at <34 weeks’ gestation received at least one dose of ACS but only 55.5% received their first dose in the 7 days preceding birth. Additionally, nearly one in five of all pregnancies given antenatal steroids delivered at term. Over the study period, steroid use significantly increased both for preterm births and for babies ultimately born at term. This was driven by increasing steroid use in the late preterm period, likely reflecting adoption of guidelines in the United States supporting late preterm steroid administration [16, 17]. The preterm birth rate, steroid use, and timing in early preterm births remained stable over the study period. Optimal timing of the first steroid course in early preterm births was positively associated with nulliparity and preeclampsia, and negatively associated with placenta previa and with ruptured membranes.

Published data on the timing of steroids in the United States are limited, and the use of inconsistent definitions of “optimal timing” makes direct study comparisons challenging. One study of 2259 births from two cohorts reported optimally timed steroids (defined as 2–7 days before delivery) in 36.3% of early preterm births [18]. Another study of 23,671 early preterm neonates from 276 neonatal intensive care units reported 87.6% received steroids and 66.9% within 7 days of birth [19]. Use of standard measures for antenatal steroids, such as the SMFM quality metrics used in our study, should improve future benchmarking and evaluations [8].

Our observations about the timing of ACS in relation to birth are important for two main reasons. First, in early preterm births (<34 weeks’ gestation), the greatest evidence supporting steroid efficacy is when they are given between 12 h and 7 days before birth, with limited evidence supporting benefit as long as 2 weeks after the first dose [18, 19, 20]. In our cohort, many early preterm births received either no steroids or received them outside of what is considered to be the optimal 7‐day window. This finding was static over the 5‐year study period. Given that early preterm births benefit most from antenatal steroids, greater efforts are needed to understand and improve the timing of antenatal steroids in this group, though this might be challenging. Our exploratory analysis highlighted some factors associated with a lower rate of optimal timing of steroids, which could be areas of focus for improvement efforts.

Second, we observed an increase in antenatal steroid use overall in our cohort and in pregnancies that ultimately delivered at term over the study period. Studies using US birth certificate data have also documented an increase in antenatal steroid use since publication of the ALPS trial in 2016 [4, 5, 6]. Freret et al. [6] reported steroid exposure in 0.77% of term births in 2017 (up from 0.43% in 2015). However, our study suggests that steroid exposure in term births might be higher: 0.96% in 2017 and 1.57% in 2021. In our cohort, the increase in steroid exposure in term births was driven by increased late preterm steroid administration, which accounted for approximately 40% of all steroid use in 2021 (Appendix S1 and Table S3).

While there is evidence of improved neonatal respiratory outcomes following late preterm steroids [7], there have also been observational studies reporting associations with an increased risk of infectious diseases in the first 4 years of life and with increased concerns for neurocognitive impairment in term or late preterm births [21, 22, 23, 24]. However, retrospective studies can have limitations including the potential for collinearity bias where the indication for giving antenatal steroids (e.g., severe growth restriction) might also be associated with an increased risk for the outcome (e.g., childhood neurocognitive impairment) [25]. A systematic review and meta‐analysis that included randomized controlled trials and quai‐experimental studies, considered at low‐to‐moderate risk of bias, found no consistent association between antenatal steroids and neurocognitive impairment in children [26]. The largest randomized controlled trial with long‐term follow‐up that was included in this meta‐analysis was the ALPS trial. This reported no evidence of neurocognitive impairment in children randomized to antenatal steroids but only included follow‐up on approximately 1/3 of participants [27].

Acknowledging the limitations of current evidence, in light of concerns raised about a potential association between the long‐term effects of antenatal steroids in late preterm and term births, it seems prudent to be judicious about antenatal steroid use at later gestational ages. The risk of neonatal respiratory complications diminishes with increasing gestational age, but the ALPS trial was not powered to evaluate antenatal steroids by gestational week over the late preterm period [27]. However, an analysis of the ALPS trial data published in 2024 highlighted the variation in absolute risk reduction of antenatal steroids in the late preterm period by gestational age and planned mode of delivery, suggesting that an individualized approach to late preterm steroid use might be most appropriate for shared decision making [28].

Strengths of our study include the use of granular clinical data and sociodemographic information, as well as data on the timing of steroid administration. Our study included hospitals with different levels of care and, therefore, different risk profiles of their patient populations. A multilevel modeling approach accounted for clustering within hospitals. However, there are limitations to our study. Our study was performed in one region, so findings may not be generalizable to the entire United States or globally. In addition, the data on repeat courses of steroids were limited and the variables available were restricted to those collected by the collaborative. As this is an observational study, residual confounding cannot be excluded as a cause of observed associations. There may be important unmeasured factors that could cause this confounding; in particular, clinical data may not directly capture clinician decision‐making processes nor fully capture clinical nuances such as indications and contraindications for ACS use. The data used for this study undergo validation checks against the clinical record from which the data are abstracted; however, it is not possible to validate the accuracy of the clinical record itself. While we expect the clinical record to be highly accurate with respect to data that are important for clinical decision making, the accuracy of non‐clinical factors such as race/ethnicity may be lower and is difficult to measure. Timing of ACS was only to the calendar day, limiting the precision of the steroid to delivery time, which in particular did not allow us to evaluate short intervals.

## 5 CONCLUSION

Our study reports high steroid use in early preterm births, but less optimal timing of the first dose of steroids, highlighting a need for better prediction of preterm birth and for quality improvement efforts to focus on timing, as well as the use of ACS. Increasing use of steroids was observed in late preterm and term births, supporting the need for further long‐term follow‐up studies to evaluate children exposed to steroids later in pregnancy. Using robust causal methods, such studies will be key to appropriately reflect on current practices and guidelines.

## CONFLICT OF INTEREST STATEMENT

The authors report no conflicts of interest.

## ETHICS STATEMENT

The University of Glasgow College of Medical, Veterinary & Life Sciences (MVLS) Ethics Committee approved the study (Study number 200220401).

## Supporting information



Supporting Information

## Data Availability

The data that support the findings of this study are available from the Foundation for Healthcare Quality's Obstetrical Care Outcomes Assessment Program (OB COAP), an ongoing perinatal quality improvement collaborative. Restrictions apply to the availability of these data, which were collected for quality improvement purposes and used under license for this study. Questions about the availability of the data may be directed to fhcq@qualityhealth.org.
