# Supplementary material for: An evaluation of antenatal corticosteroid use and timing in a large US birth cohort 2017–2021
Source: Pregnancy (Hoboken). 2026 Apr 7;2(3):e70287. doi: 10.1002/pmf2.70287 (PMC13344416; doi:10.1002/pmf2.70287)
Supplement: Supplementary file 1 [file PMF2-2-e70287-s001.docx]

**Supplementary Digital Content**

**Appendix 1 Supplementary tables**

**Supplementary Table 1** Percentage of pregnant people with term livebirths who had antenatal steroids at some point in the pregnancy, stratified by year of delivery.

| **Year** | **Term livebirths that received antenatal steroids <37 weeks**  **% (n/N)** |
| --- | --- |
| 2017 | 0.96 (187/19,537) |
| 2018 | 1.22 (238/19,505) |
| 2019 | 1.11 (205/18,509) |
| 2020 | 1.42 (267/18,509) |
| 2021 | 1.57 (307/19,559) |
| Pearson Chi2 | p<0.001 |
| All years | 1.26 (1,204/95,872) |

**Supplementary Table 2** Timing of delivery in pregnancies given antenatal corticosteroids (ACS) before 34 weeks’ gestation.

| **Timing of delivery in pregnancies given ACS prior to 34 weeks’ gestation (N=3,988) n (%)** | |
| --- | --- |
| <34 weeks | 2212 (55.5) |
| 34^+0^ -36^+6^ weeks | 1088 (27.3) |
| >=37 weeks | 688 (17.3) |

**Supplementary Table 3** Proportion of antenatal corticosteroids by gestational age at administration of the first dose, stratified by year.

|  | **Gestational week at ACS administration** | | | | |
| --- | --- | --- | --- | --- | --- |
| **Total ACS** | **22^+0^-23^+6^w**  n, (%) | **24^+0^-33^+6^w**  n, (%) | **34w**  n, (%) | **35w**  n, (%) | **36w**  n, (%) |
| 2017 (n=1,207) | 33 (2.7) | 789 (65.4) | 160 (13.3) | 138 (11.4) | 87 (7.2) |
| 2018 (n=1,267) | 25 (2.0) | 769 (60.7) | 186 (14.7) | 169 (13.3) | 118 (9.3) |
| 2019 (n=1,234) | 35 (2.8) | 752 (60.9) | 187 (15.3) | 142 (11.5) | 123 (10.0) |
| 2020 (n=1,346) | 34 (2.5) | 746 (55.4) | 212 (15.8) | 209 (15.5) | 145 (10.8) |
| 2021 (n=1,389) | 32 (2.3) | 778 (56.0) | 229 (16.5) | 221 (15.9) | 129 (9.3) |
| **All (6,443)** | **154 (2.4)** | **3,834 (59.5)** | **974 (15.1)** | **879 (13.6)** | **602 (9.3)** |

**Supplementary Table 4** The proportion of singleton pregnancies without pre-pregnancy diabetes that delivered at 34^+0^-36^+6^ weeks gestation that received antenatal corticosteroids stratified by year.

| **Year** | **% (n) of live births at 34^+0^-36^+6^ weeks’ gestation given steroids** | Odds Ratio  (95% CI) | **% (n) of live births at 34^+0^-36^+6^ weeks’ gestation given steroids**  **within 7 days** | Odds Ratio  (95% CI) |
| --- | --- | --- | --- | --- |
| 2017 (N=1,197) | 36.3% (434) | Reference | 24.6% (295) | Reference |
| 2018 (N=1,205) | 40.3% (486) | 1.19  (1.01,1.40) | 27.6% (333) | 1.17  (0.97, 1.40) |
| 2019 (N=1,189) | 41.0% (487) | 1.21  (1.03, 1.44) | 26.8% (319) | 1.12  (0.93, 1.35) |
| 2020 (N=1,176) | 49.2% (576) | 1.69  (1.43, 1.99) | 33.3% (392) | 1.53  (1.28, 1.83) |
| 2021 (N=1,233) | 46.0% (567) | 1.50  (1.27, 1.76) | 31.1% (383) | 1.38  (1.15, 1.65) |
| All (N=6,000) | 42.5% (2,550) | - | 28.7% (1,722) | - |

**Supplementary Table 4** Optimally-timed antenatal corticosteroids (defined as given within 7 days) versus no or suboptimal ACS in early preterm live births (<34 weeks’ gestation) stratified by characteristics within the study cohort.

|  | **No ACS or ACS more than 7 days before early preterm livebirth**  N=1,175  n (%) | **ACS within 7 days of early preterm live birth**  N=1,466  n (%) | **Pearson chi2 test**  **p-value** |
| --- | --- | --- | --- |
| **Maternal age, years** |  |  |  |
| <20 (n=114) | 51 (44.7) | 63 (55.3) |  |
| 20 -24 (n=346) | 138 (39.9) | 208 (60.1) |  |
| 25-29 (n=692) | 314 (45.4) | 378 (54.6) |  |
| 30-34 (n=761) | 330 (43.4) | 431 (56.6) |  |
| 35-39 (n=580) | 269 (46.4) | 311 (53.6) |  |
| 40-54 (n=147) | 72 (49.0) | 75 (51.0) |  |
| Missing (n=1) | 1 (100) | 0 |  |
| **Final BMI, (kg/m2)** |  |  | P=0.162 |
| <25 (n=345) | 171 (48.0) | 185 (52.0) |  |
| 25-29 (n=402) | 311 (43.7) | 401 (56.3) |  |
| >=30 (n=795) | 607 (43.3) | 795 (56.7) |  |
| Missing (n=171) | 86 (50.3) | 85 (49.7) |  |
| **Pregnancy history** |  |  | P<0.001 |
| Nulliparous (n=1,176) | 471 (40.1) | 705 (60.0) |  |
| Parous, *no* previous term births (n=1,197) | 574 (48.0) | 623 (52.1) |  |
| Parous, previous term births (n=268) | 130 (48.5) | 138 (51.5) |  |
| **Health history** |  |  |  |
| Pre-pregnancy diabetes, |  |  | 0.455 |
| Yes (n=184) | 77 (41.9) | 107 (58.2) |  |
| No (n=2,457) | 1,098 (44.7) | 1,359 (55.3) |  |
| Pre-pregnancy hypertension, n (%) |  |  | 0.208 |
| Yes (n=399) | 166 (41.6) | 233 (58.4) |  |
| No (n=2,242) | 1,009 (45.0) | 1,233 (55.0) |  |
| **Pregnancy complications** |  |  |  |
| Multiple pregnancy |  |  | 0.564 |
| Yes (n=435) | 199 (45.8) | 236 (54.3) |  |
| No (n=2,206) | 976 (44.2) | 1,230 (55.8) |  |
| Preeclampsia/gestational hypertension (irrespective of gestational age at onset) |  |  | <0.001 |
| Yes (n=819) | 285 (34.8) | 534 (65.2) |  |
| No (n=1,817) | 885 (48.7) | 932 (51.3) |  |
| Missing | 5 | 0 |  |
| Placenta previa |  |  | <0.001 |
| Yes (n=62) | 45 (72.6) | 17 (27.4) |  |
| No (n=2,579) | 1,130 (43.8) | 1,449 (56.2) |  |
| Fetal abnormality |  |  | 0.094 |
| Yes (n=155) | 79 (51.0) | 76 (49.0) |  |
| No (n=2,486) | 1,096 (44.1) | 1,390 (55.9) |  |
| Membranes ruptured at admission to labor and delivery |  |  | P<0.001 |
| Yes (n=871) | 433 (49.7) | 438 (50.3) |  |
| No (n=1,740) | 726 (41.7) | 1,014 (58.3) |  |
| Missing (n=30) | 16 (53.3) | 14 (46.7) |  |
| Any smoking during pregnancy |  |  | 0.030 |
| Yes (n=277) | 140 (50.5) | 137 (49.5) |  |
| No (n=2361) | 1,032 (43.7) | 1,329 (56.3) |  |
| Missing (n=3) | 3 (100) | 0 |  |
| Illicit substance use during pregnancy |  |  | 0.003 |
| Yes (n=186) | 102 (54.8) | 84 (45.2) |  |
| No (n=2,455) | 1,073 (43.7) | 1,382 (56.3) |  |
| **Sociodemographic characteristics** |  |  |  |
| Race |  |  | 0.115 |
| Hispanic or Latina (n=460) | 209 (45.4) | 251 (54.4) |  |
| Non-Hispanic Asian (n=281) | 119 (42.4) | 162 (57.7) |  |
| Non-Hispanic Black  (n=243) | 104 (42.8) | 139 (57.2) |  |
| Non-Hispanic Hawaiian or Pacific Islander (n=58) | 31 (53.5) | 27 (46.6) |  |
| Non-Hispanic Native American or Native Alaskan (n=68) | 36 (52.9) | 32 (47.1) |  |
| Non-Hispanic White  (N=1,228) | 558 (45.4) | 670 (54.6) |  |
| Multiple racial groups or group not included above. | 20 (30.3) | 46 (69.7) |  |
| Missing, % (n=237) | 98 (41.4) | 139 (58.7) |  |
| Distressed communities index (quintiles) |  |  | 0.304 |
| Prosperous (n=741) | 330 (44.5) | 411 (55.5) |  |
| Comfortable (n=732) | 326 (44.5) | 406 (55.5) |  |
| Mid-tier (n=445) | 179 (40.2) | 266 (59.8) |  |
| At risk (n=485) | 223 (46.0) | 262 (54.0) |  |
| Distressed (n=182) | 88 (48.4) | 94 (51.7) |  |
| Missing (n=56) | 29 (51.8) | 27 (48.2) |  |
| Commercial payer (n=1,312) | 558 (42.5) | 754 (57.5) | 0.059 |
| Other type of payer (n=1,311) | 606 (46.2) | 705 (53.8) |  |
| Missing (n=18) | 11 (61.1) | 7 (38.9) |  |
| **Pregnancy care** |  |  |  |
| Intrapartum transfer into hospital |  |  | P<0.001 |
| Yes (n=569) | 155 (27.2) | 414 (72.7) |  |
| No (n=2072) | 1,020 (49.2) | 1,052 (50.8) |  |
| Prenatal care professional n (%) |  |  | P=0.011 |
| Family practitioner (n=105) | 43 (41.0) | 62 (59.1) |  |
| Maternal fetal medicine specialist (n=419) | 217 (51.8) | 202 (48.2) |  |
| Midwife (n=112) | 45 (40.2) | 67 (59.8) |  |
| Obstetrician (n=1,377) | 612 (44.4) | 765 (55.6) |  |
| Other (n=397) | 156 (39.3) | 241 (60.7) |  |
| Missing, n=2,641 | 102 (44.2) | 129 (55.8) |  |
| Hospital level of neonatal care, n (%) |  |  | <0.001 |
| I (n=32) | 24 (75.0) | 8 (25.0) |  |
| II (n=222) | 112 (50.4) | 110 (49.5) |  |
| III-IV (n=2,387) | 1,039 (43.5) | 1,348 (56.5) |  |
